# Matrix metalloproteinase 9 contributes to gut microbe homeostasis in a model of infectious colitis

**DOI:** 10.1186/1471-2180-12-105

**Published:** 2012-06-13

**Authors:** David M Rodrigues, Andrew J Sousa, Steve P Hawley, Linda Vong, Melanie G Gareau, Sachin A Kumar, Kathene C Johnson-Henry, Philip M Sherman

**Affiliations:** 1 Department of Paediatrics, University of Toronto, 27 Kings College Circle, Toronto, ON, M5S 1A1, Canada; 2 Cell Biology Program, Hospital for Sick Children, Room 8409 555 University Avenue, Toronto, ON, M5G 1X8, Canada

## Abstract

**Background:**

Inflammatory bowel diseases are associated with increased expression of zinc-dependent Matrix Metalloproteinase 9 (MMP-9). A stark dysregulation of intestinal mucosal homeostasis has been observed in patients with chronic inflammatory bowel diseases. We therefore sought to determine the contribution of MMP-9 to the pathogenesis of *Citrobacter rodentium*-induced colitis and its effects on gut microbiome homeostasis.

**Results:**

Wild-type and MMP-9^−/−^ mice aged 5–6 weeks were challenged with *C. rodentium* by orogastric gavage and sacrificed either 10 or 30 days post-infection. Disease severity was assessed by histological analysis of colonic epithelial hyperplasia and by using an *in vivo* intestinal permeability assay. Changes in the inflammatory responses were measured by using qPCR, and the composition of the fecal microbiome evaluated with both qPCR and terminal restriction fragment length polymorphism. Activation and localization of MMP-9 to the apical surface of the colonic epithelium in response to *C. rodentium* infection was demonstrated by both zymography and immunocytochemistry. The pro-inflammatory response to infection, including colonic epithelial cell hyperplasia and barrier dysfunction, was similar, irrespective of genotype. Nonmetric multidimensional scaling of terminal restriction fragments revealed a different fecal microbiome composition and *C. rodentium* colonization pattern between genotypes, with MMP-9^−/−^ having elevated levels of protective segmented filamentous bacteria and interleukin-17, and lower levels of *C. rodentium*. MMP-9^−/−^ but not wild-type mice were also protected from reductions in fecal microbial diversity in response to the bacterial enteric infection.

**Conclusions:**

These results demonstrate that MMP-9 expression in the colon causes alterations in the fecal microbiome and has an impact on the pathogenesis of bacterial-induced colitis in mice.

## Background

Microbe-microbe and host-microbe interactions combine to maintain intestinal homeostasis and proper functioning of the gut, including immunomodulation and intestinal epithelial barrier function [1]. The contribution of specific interactions, including cooperation and competition at the microbe-microbe level, is still not well characterized. However, an *in vivo* mesocosm model has revealed the dynamics of a simple gut microbial community consisting of *Lactobacillus johnsoniiBifidobacterium longum* and *Escherichia coli*[2]. Host-microbe interactions have been studied more intensely under both physiological and pathological conditions, including the contribution of mucins, antimicrobial peptides and secretory antibodies in maintaining gut homeostasis. In healthy individuals, these interactions combine to produce a fecal microbiota of notable stability [3] that is in stark contrast to the dysregulation of intestinal mucosal homeostasis observed in patients with chronic inflammatory bowel diseases (IBD) [1]. Through analysis of the fecal microbiota in patients with Crohn disease, a microbial signature has been described for the disease state, compared to unaffected relatives [4].

There is evidence that the chronic consequences of enterohemorrhagic *Escherichia coli* (EHEC) serotype O157:H7 infection, which causes bloody diarrhea and the haemolytic uremic syndrome [5]**,** include intestinal dysbiosis which then contributes to the chronic symptoms that characterize post-infectious irritable bowel syndrome (IBS) [6] and chronic IBD [7]. *Citrobacter rodentium* is a murine-specific enteric pathogen genetically related to EHEC that is capable of causing similar dysregulation of intestinal mucosal homeostasis in a mouse model of colitis. Infection with *C. rodentium* results in a decrease in microbial diversity and an inflammatory response in the colon of infected mice [8]. Pathogenicity of both EHEC and *C. rodentium* is attributed to locus of enterocyte effacement (LEE) and non-LEE type III effector proteins, which mediate host responses to infection. The host response to infection is characterized by increases in T helper (T_H_)-1 and T_H_-17 cells, colonic epithelial cell hyperplasia and mucosal barrier dysfunction [9].

The matrix metalloproteinase (MMP) family consists of 24 zinc-dependent proteases, which are secreted as inactive zymogens by many cell types including proinflammatory cells, fibroblasts and epithelial cells. Increased expression of MMPs −1, -2, -3, -8, -9, and −12 each have been associated with IBD [10-12]. Individual MMPs vary in substrate specificity, and may have multiple substrates for which they are biologically active. These proteases are involved in multiple biological processes, including extracellular matrix remodeling [13], protein maturation [14] and bactericidal activity [15].

Other proteases are also implicated in the establishment of infectious colitis, as serine protease inhibitors can lessen the severity of *C. rodentium*-induced colitis [16]. In other animal models of IBD, MMP-9 is indispensible for establishment of inflammation in the dextran sodium sulphate (DSS) colitis model [17] through suppression of epithelial wound healing and goblet cell differentiation [18]. However, relationships between disease severity, the activation of specific MMPs and alterations in gut microbial diversity have not been fully determined. Therefore, the aim of this study was to determine the contribution of MMP-9 to the pathogenesis of *C. rodentium* infection and its influence on microbial diversity in the gut.

## Results

### MMP-9 is upregulated in the colon of wild-type mice 10 days post infection with *C. rodentium* and localizes at the apical surface of the colonic epithelium

**Figure 1:**
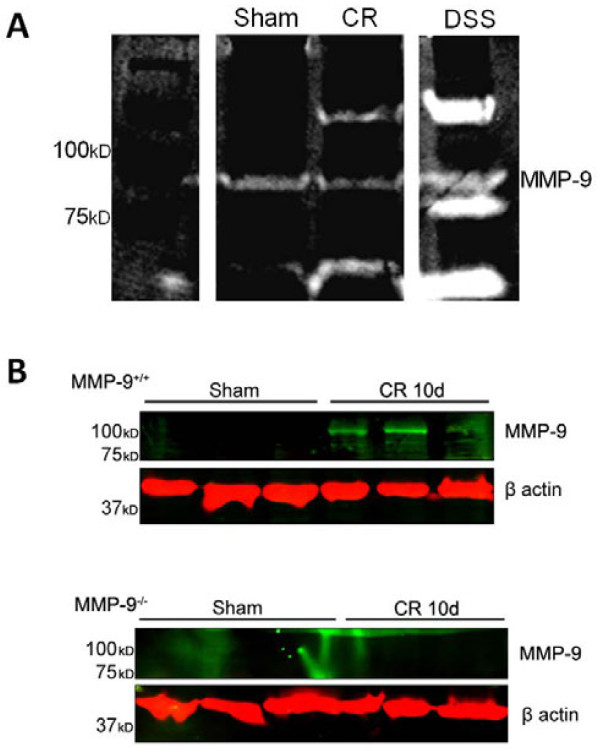
***C. rodentium *infection is associated with increased MMP-9 activation.** (**A**) Representative gelatin zymogram showing the absence of MMP-9 activity in sham-infected animals and activation of MMP-9 at 10d PI with *C. rodentium*. Positive controls for MMP-9 were obtained from colonic homogenates from dextran sodium sulphate (DSS)-treated animals, at the peak of inflammation (8d post-DSS). (**B**) Representative western blot demonstrates increased activation of MMP-9 (92 kDa) in whole colon homogenates obtained from *C. rodentium*-infected WT and MMP-9^−/−^ mice at 10 days PI, compared to sham-infected mice.

To determine whether MMP-9 was involved in the pathogenesis of *C. rodentium* infection, protein expression and bioactivity were assessed in whole colon homogenates obtained from both uninfected and infected mice. Gelatin zymography was utilized to determine if MMP-9 was able to cleave gelatin, a physiological substrate of this protease [19]. Zymographic analysis revealed a band of gelatin digestion at 92kD in colon homogenates from mice 10 days after challenge with *C. rodentium* (Figure 1A)*,* which was comparable to a positive control used for MMP-9 activity (DSS-treated mouse colon). The band was absent in zymograms renatured and incubated with 20 mM EDTA, reinforcing that this band is a metalloprotease (data not shown). Taken together, these data show that bioactive MMP-9 is not expressed normally in mouse colon, but protease expression is upregulated in response to an infectious colitis. In addition, immunoblotting revealed the presence of a 92kD MMP-9 immunoreactive band in the infected samples that was undetectable in both uninfected controls and infected MMP-9^−/−^ mice (Figure 1B).

### MMP-9^−/−^ and wild-type *C. rodentium*-infected mice display similar colonic epithelial hyperplastic responses and changes in barrier dysfunction

**Figure 2:**
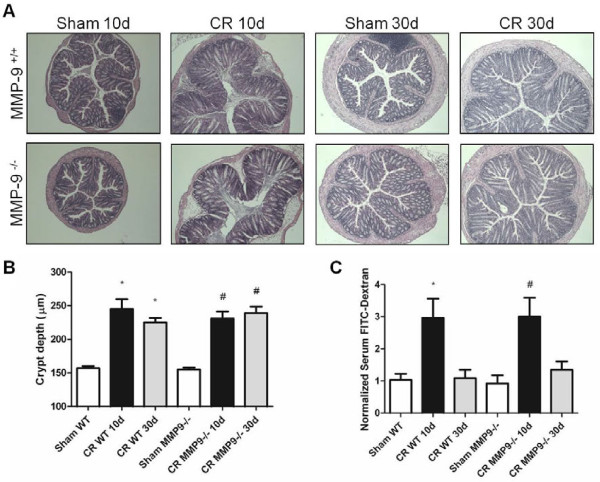
**MMP-9**^**−/−**^**and WT mice infected with*C. rodentium*have similar histopathology and mucosal barrier dysfunction.** (**A**) Representative H & E stained images of colonic tissues demonstrating *C. rodentium*-induced inflammation in MMP-9^+/+^ and MMP-9^−/−^ mice. Scale bar, 100 μm. (**B**) Quantitative analysis shows significant increases in colonic crypt height in MMP-9^+/+^ and MMP-9^**−/−**^ mice at 10d and 30d PI, compared to sham-infected mice. N = 12-18. (**C**) Fluorometric analysis of a 4 kDa FITC-dextran probe in serum samples obtained from WT and MMP-9^−/−^ mice in the presence or absence of *C. rodentium* infection (10d and 30d PI). *P<0.05 compared to Sham WT; ^#^*P*<0.05 compared to Sham MMP-9^−/−^. N = 7-17.

MMP-9^−/−^ mice were used to determine a possible contribution of MMP-9 in the pathogenesis of *C. rodentium*-infection. Both wild-type (WT) and MMP-9^−/−^ mice demonstrated hyperplastic responses to *C. rodentium* at 10 days post infection (PI) **(**Figure 2A), with the degree of hyperplasia comparable between the two groups during this peak phase of inflammation (Figure 2B) (*P* > 0.05). At 30 days PI, when the overt inflammatory response has ceased [9,20], epithelial hyperplasia remained elevated in both groups of infected mice (*P* < 0.05).

To investigate the presence of deficits in epithelial barrier function, WT and MMP-9^−/−^ mice were orogastrically gavaged with FITC-labeled dextran probe (4 kDa). Although dextran flux does not localize the source of macro-molecular uptake along the length of the gastrointestinal tract, the probe is routinely used as an indicator of gut permeability in animal models [21]. Plasma concentrations of the probe were then determined by fluorimetry and used as an indication of intestinal permeability, as described previously [22]. Significant increases in intestinal barrier dysfunction were detected, compared to sham-infected mice, when WT (10d PI) and MMP-9^−/−^ (10d PI) mice were infected with *C. rodentium* (Figure 2C) (*P* < 0.05). However, there were no differences noted between WT and MMP^−/−^ infected groups at 10d PI. At 30d PI, intestinal permeability had returned to baseline levels in both WT and MMP-9^−/−^ mice.

**Figure 3:**
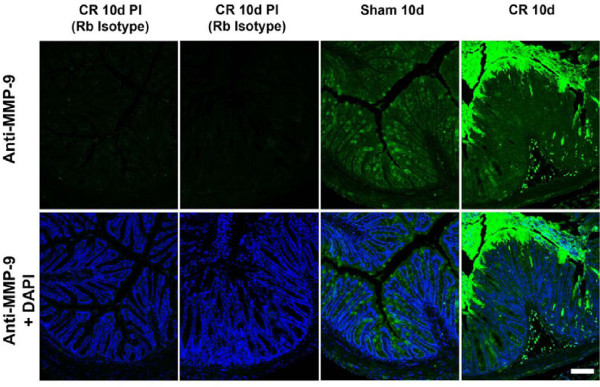
**MMP-9 expression is increased with*C. rodentium*infection.** Immunohistochemistry shows that MMP-9 distributed throughout the crypts (green) in uninfected WT mice is localized primarily to the apical surface of intestinal epithelium in *C. rodentium*-infected (10d) WT mice. Scale bar, 100 μm.

Immunocytochemistry of sham and *C. rodentium*-infected (10d) colon from WT mice revealed localized expression of MMP-9 (green) primarily at the apical surface of intestinal epithelium, with more intense staining in infected mice (Figure 3). No non-specific binding of anti-MMP-9 antibody was observed in isotype controls.

### *C. rodentium* infection modulates goblet cells in colonocytes

**Figure 4:**
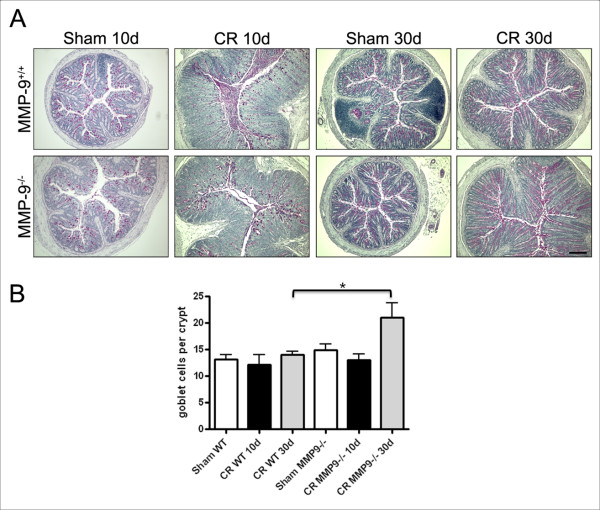
**Post-infectious goblet cell hyperplasia occurs in MMP**-9^−/−^**mice.** (**A**) Representative histology demonstrating goblet cells stained positive (red) for PAS in MMP-9^+/+^ and MMP-9^−/−^ colonocytes. (**B**) Quantitative analysis shows similar numbers of goblet cells in WT and MMP-9^−/−^ mice at 10d PI. A significant increase in goblet cells was observed in MMP-9^−/−^ mice at 30d PI. *P<0.05 compared to WT-infected animals. N = 3–5.

Periodic Acid Shiff (PAS) staining was used to assess the qualitative (Figure 4A) and quantitative (Figure 4B) changes to goblet cells that occurred during *C. rodentium* infection. There were no differences in the number of positively stained red cells in colonic crypts from MMP-9^+/+^ cells and MMP-9^−/−^ mice at 10d PI. Quantitative analysis of the number of positive PAS stained cells per crypt showed a significant increase in MMP-9^−/−^ mice at 30d PI, compared to wild type infected mice (*P* < 0.05).

### MMP-9^−/−^ mice have elevated mRNA levels of interleukin (IL)-17

**Figure 5:**
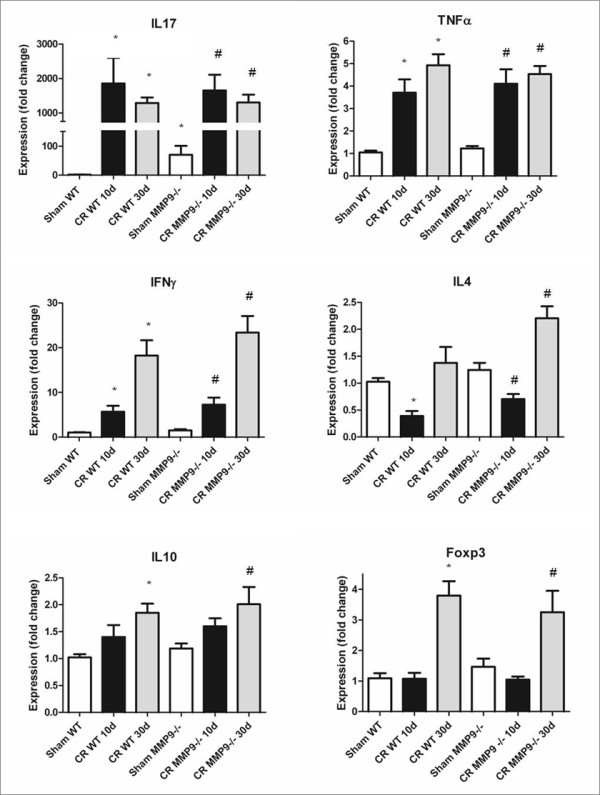
**MMP-9**^**−/−**^**mice demonstrate elevated baseline IL-17 transcription, compared to WT mice.** Analysis of mRNA from whole-thickness distal colons obtained from infected and uninfected WT and MMP-9^−/−^ mice for the following genes: IL-17, TNFα, IFNγ, IL-4, IL-10, FOXP3 and β–actin (housekeeping gene). *P<0.05 compared to Sham WT; #*P*<0.05 compared to Sham MMP-9^−/−^. N = 6-18.

To delineate the impact of MMP-9 deficiency on adaptive immune responses to *C. rodentium*, qPCR was employed to measure the transcription of various pro- and anti-inflammatory cytokines. Uninfected MMP-9^−/−^ mice had higher mRNA levels of IL-17 than WT animals (*P* < 0.05) (Figure 5), but not TNFα, IFNγ, IL-4, IL-10 and FOXP3 (*P*>0.05). At 10 and 30 days PI, mice had significant increases in IL-17, TNFα and IFNγ (for all *P* < 0.05), but levels did not differ between MMP-9^−/−^ and WT mice (*P*>0.05). At 30 days PI, both groups of mice demonstrated elevated IL-10 and FOXP3 mRNA (for both *P* < 0.05), indicating the resolution phase of the infectious colitis.

### The gut microbiome is altered in MMP-9^−/−^ mice

**Figure 6:**
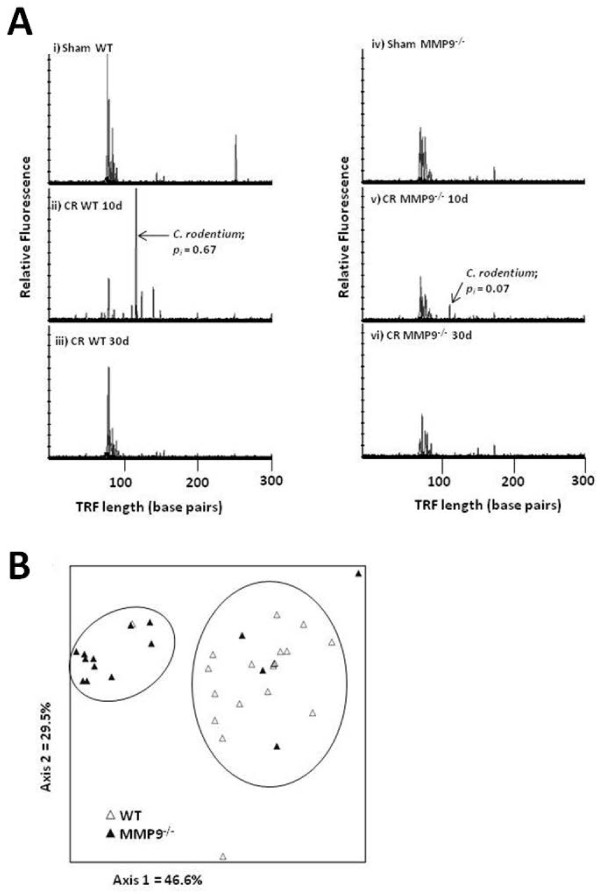
**MMP-9**^**−/−**^**mice have an altered intestinal microbiome and decreased*C. rodentium*colonization efficiency.** (**A**) T-RFLP was employed to track the colonization of *C. rodentium* in infected mice by following the presence and intensity of the 118 bp peak on electropherograms (indicated by arrows). (**B**) Nonmetric multidimensional scaling of terminal restriction fragments from WT and MMP-9^−/−^ mice reveals two distinct microbial communities. N = 15-18.

Variations in the proportion of *C. rodentium* in fecal samples were represented in electropherograms with each of the graphs signifying one mouse. *C*. *rodentium* was identified in WT (*p*_*i*_ = 0.67) and MMP-9^−/−^ mice (*p*_*i*_ = 0.07) at 10 days PI and undetectable at 30 days PI (Figure 6A) [9]. This observation prompted an evaluation and comparison of the bacterial composition in stool pellets obtained both before and after the enteric infection. Peaks from each of the electropherograms generated were analysed by nonmetric multidimensional scaling (NMS) to screen for microbial community differences between the WT and MMP-9 gene knockout mice (Figure 6B). Multi-response permutation procedure (MRPP) of NMS scores revealed significantly different bacterial communities between WT and MMP-9^−/−^ mice (Table 1). Pair-wise comparisons between experimental groups also revealed that the microbiota of sham infected WT mice differed from that of the *C. rodentium*-infected WT 10 day group, while no significant changes were observed between sham infected MMP-9^−/−^ and *C. rodentium*-infected mice. In addition, all other comparison groups remained unchanged (Table 1).

**Table 1:** **Multi-response permutation procedure (MRPP) analysis of wild type (WT) and MMP-9**^**−/−**^**mice in the absence (Sham) and presence of an enteric bacterial pathogen,** ***C. rodentium*** **(CR)**

**Experimental group**	***p*-value**	**Chance-corrected within-group agreement (A)**
Sham WT vs. Sham MMP-9^−/−^	**0.00003**	**0.1739**
Sham WT vs. CR WT 10d	**0.0039**	**0.2449**
Sham WT vs. CR WT 30d	0.0933	0.0579
CR WT 10d vs. CR WT 30d	0.0643	0.0824
Sham MMP-9^−/−^ vs. CR MMP-9^−/−^ 10d	0.1235	0.1020
Sham MMP-9^−/−^ vs. CR MMP-9^−/−^ 30d	0.3164	0.0121
CR MMP-9^−/−^ 10d vs. CR MMP-9^−/−^ 30d	0.3192	0.0149

N = 3-8 in each experimental group.

**Figure 7:**
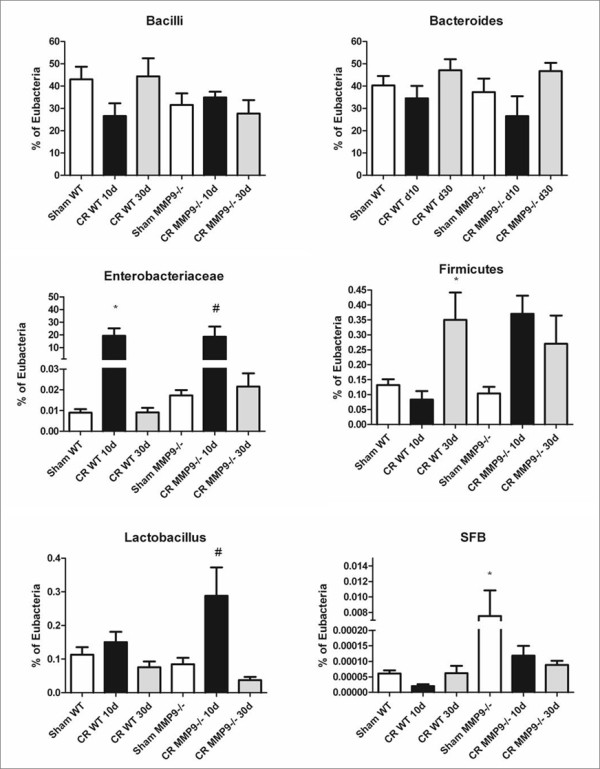
**MMP-9**^**−/−**^**mice have a microbiome enriched in segmented filamentous bacteria.** qPCR analysis of bacterial 16 s rRNA sequences specific to the following communities of bacteria: Bacillus, Bacteroides, Enterobacteriaceae, Firmicutes, Lactobacilli/Lactococci**,** and SFB (“*A immunis*”).*P<0.05 compared to Sham WT; #*P*<0.05 compared to Sham MMP-9^−/−^. N = 4-11.

Infection of WT mice with *C. rodentium* resulted in a lower Shannon diversity index (indicative of a less diverse bacterial population) and decreased evenness (reflecting an increase in the dominance of a phylotype) relative to Sham WT, affirming that *C. rodentium* became a major component of the detectable gut microbiota (Table 2). This correlates with the significant rise in Enterobacteriaceae in mice 10d PI with *C. rodentium* (Figure 7). Contrary to what was seen with WT mice, MMP-9^*−/−*^ mice infected with *C. rodentium* showed no significant change in the Shannon diversity index at 10d and 30d PI. A more even spread of phylotypes (higher evenness; decrease in the dominance of *C. rodentium*), was observed in MMP-9^−/−^ mice at both 10d and 30d PI compared to Sham MMP9^−/−^ (Table 2).

**Table 2:** **Shannon diversity index and measurement of Evenness of the fecal microflora prior to and after challenge with** ***C. rodentium*** **(CR, in wild type (WT) and MMP-9 gene knockout mice**

**Experimental group**	**Shannon-seiner diversity**	**Evenness**
Sham WT	1.88 ± 0.10	0.81 ± 0.02
CR WT 10d	1.32 ± 0.14*	0.65 ± 0.06*
CR WT 30d	1.67 ± 0.08	0.80 ± 0.02
Sham MMP-9^−/−^	1.59 ± 0.05	0.81 ± 0.01
CR MMP-9^−/−^ 10d	1.83 ± 0.10	**0.87 ± 0.03**^**Ψ**^
CR MMP-9^−/−^ 30d	1.70 ± 0.09	**0.91 ± 0.01**^**Ψ**^

N = 3-8 in each experimental group

* p < 0.05 vs WT uninfected and WT 30 days PI

**Ψ** p < 0.05 vs MMP-9^−/−^ uninfected

qPCR analysis of stool samples from uninfected animals showed no marked differences in levels of Bacilli, Bacteroides, Enterobacteriaceae, Firmicutes or Lactobacilli between uninfected WT and MMP-9^−/−^ mice (Figure 7). However there was a larger population of segmented filamentous bacteria in MMP-9^−/−^ mice (*P* < 0.05), which have been shown to dramatically impact host adaptive immune responses to challenge with *C. rodentium*[23]. At 10 days post *C. rodentium* challenge, there was an increase in Lactobacilli in MMP-9^−/−^ mice compared to WT (*P* < 0.01). Taken together, these data show that the intestinal microbiome differs between WT and MMP-9^−/−^ mice, both before and following an infectious challenge.

## Discussion

Bioactive MMP-9 is present within the colonic epithelium and becomes localized primarily near the apical surface of the intestinal epithelium when associated with *C. rodentium* infection. Studies have shown that MMP-9 is upregulated in human IBD with acute inflammation [11,24]. Deletion of MMP-9 in animal models has proven beneficial in attenuating *S. typhimuium* and DSS-induced colonic injury and inflammation [19,25,26]. The effect of MMP-9 on the gut microbiota has not been previously evaluated. This study shows the contribution of MMP-9 in the pathobiology of *C. rodentium* infection and an impact on the composition of the fecal microbiota. We demonstrate that despite similar *C. rodentium*-induced colonic epithelial responses between WT and MMP-9^−/−^ mice, there is a different microbial composition between genotypes that results an altered microbial response following an infectious challenge. These differences were revealed by nonmetric multidimensional scaling of terminal restriction fragments. The findings indicate that a difference in genotypes plays a role in influencing the microbiome composition in uninfected mice.

A healthy gut microbiome is maintained through microbe-microbe and host-microbe interactions. An alteration in gut microbe homeostasis is associated with chronic IBD in humans [1] and with post-infectious IBS [6]. A change in the microbiome also occurs in response to infection with the murine-specific pathogen *Citrobacter rodentium*[21]. The importance of a healthy gut microbiome is also implicated in toxigenic *Clostridium difficile* infection*,* which is triggered by the loss of microbiota colonization resistance and the release of ecological niches previously unavailable following antibiotic treatment [27].

Infection with *C. rodentium* resulted in activation of MMP-9, as demonstrated by zymography of colonic tissue. The resulting pro-inflammatory response to infection, including colonic epithelial cell hyperplasia and barrier dysfunction, was similar irrespective of genotype. Taken together, these findings indicate that increased expression of colonic MMP-9 following infection with *C. rodentium* is not associated with the host pro-inflammatory immune responses to the enteric pathogen.

Elimination of various factors contributing to innate and humoral immunity can dramatically alter the gut microbiome. Specifically, TLR5-deficient mice develop a markedly different intestinal microbiome, which predisposes the animals to develop metabolic syndrome [28]. Furthermore, impaired innate immune function in T-bet^−/−^Rag1^−/−^ mice develop a microbiota which is colitogenic and transferable to WT mice by fecal transplantation [29].

MMP-9 deficiency is associated with altered goblet cell differentiation, leading to an enrichment of bactericidal mucins in the intestine of mice treated with dextran sodium sulphate and *Salmonella typhimurium*[26]. This enrichment in mucus secretion in the lumen could prove important for reducing nutrients for pathogen growth and, in turn, lead to altered microbe-microbe interactions thereby disrupting gut microbe homeostasis in MMP-9^−/−^ mice. Although there was no difference in the number of goblet cells present in the colonic crypts of WT and MMP-9^−/−^ mice at the height of infection 10d PI, delayed changes to the goblet cell population were observed by 30d PI. This indicates a fundamentally different innate response to infection between WT and MMP-9^−/−^ mice which may contribute to an atypical fecal microbiome in MMP-9^−/−^ mice. Recent evidence also indicates that MMPs regulate the intercellular expression of several key mediators of cell-cell binding including claudin-5 and occludin [30]. For instance, in the context of lung injury, the pore-forming cytotoxin α-hemolysin from *Staphylococcus aureus* upregulates the zinc-dependent metalloprotease ADAM10, resulting in cleavage of E-cadherin and disruption of intercellular tight junctions [31].

Most MMPs are secreted factors, but many of the proteases localize to cell surfaces where they associate with and regulate a variety of adhesion molecules, such as CD44 and β-integrins [32,33]. This indicates that MMPs could alter the binding efficiency of intestinal bacteria to host colonocytes, thereby altering the pathobiology of an infectious colitis. MMP-7 also affects gut microbe homeostasis through cleavage of reduced cyptdin-4 (r-Crp4), a mouse Paneth cell-derived α-defensin. In an *in vitro* model, cleavage of the peptide resulted in increased survival of *Salmonella enterica* serovar Typhimurium, *E. coli* ML35, *Staphylococcus aureus, Bifidobacterium bifidum, Bifidobacterium longum, Lactobacillus caseiBacteroides thetaiotaomicron,* and *Bacteroides vulgatus* relative to undigested r-Crp4 [34]. Therefore, the presence of MMPs in the colonic mucosa can mediate physiological parameters that impact on both gut homeostasis and host-microbe interactions. Disruption of these interactions leads to an altered microbial ecology and disease [35].

Segmented filamentous bacteria (SFB) "*Arthromitus immunis*” [36]; provides mucosal protection against *C. rodentium* infection, as well as mediates the production of the proinflammatory cytokines IL-17 and IL-22 [23]. In the present study, qPCR analysis of the fecal microbiome revealed a larger population of SFB and higher mRNA levels of IL-17 in MMP-9^−/−^ mice compared to WT controls, even under baseline conditions. “*A. immunis*” inhibits colonization of rabbit enteropathogenic *Escherichia coli* O103 and protects against subsequent disease development [37]. In this study, electropherograms showed that *C. rodentium* became a dominant component of the detectable microbiota in WT, but not MMP-9^−/−^ mice. As noted by others [37], this study shows that the presence of SFB may provide protection against *C. rodentium* colonization, although our results demonstrate that commensal SFB does not offer full protection against *C. rodentium*-induced colitis in C57BL/6 J mice. This observation emphasizes that a shift in the bacterial population does not have an all-or-none effect; rather, it produces a graded series of responses.

In previous studies, infection of C57BL/6 J mice with *C. rodentium* reduced fecal microbial diversity and evenness due to the dominance of *C. rodentium* in the gut microbiome [21]. A similar pattern was observed in the current study in WT but not MMP-9^−/−^ mice, as the fecal microbiota of the latter group had no changes in diversity following infection. Colonization of the cecal mucosa by the murine pathogen *Helicobacter hepaticus* also reduces microbial diversity [38].

The distinct and stable fecal microbiome in MMP-9^−/−^ mice identified in this study emphasizes that the presence of MMP-9 in mouse colon supports a microbiome that is more susceptible to *C. rodentium* colonization and reductions in microbial diversity. Given that MMP-9^−/−^ (B6.FVB(Cg)-*Mmp9*^*tm1Tvu*^/J) mice have a microbiota that is more resistant to *C. rodentium* colonization, this genotype should prove useful for future studies evaluating the contribution of microbe-microbe interactions to the pathogenesis of *C. rodentium* infection and the maintenance of microbial diversity. The role of other MMPs in maintaining the fecal microbiota upon infectious challenge will also prove to be of interest in future experimental studies.

## Conclusions

Microbe-microbe and host-microbe interactions are essential for maintaining gut health [1]. Although studies have shown that expression of matrix metalloproteinase 9 is associated with IBD, the influence of MMP-9 expression on gut microbial community dynamics has not been studied *in vivo*. This work demonstrates that, in a model of bacterial-induced colitis, the particular microbial community of MMP-9^−/−^ mice contributes to reduced levels of *C. rodentium* preventing a reduction in the microbial diversity associated with infection [21]. An altered intestinal ecosystem may lead to changes in some of the protective, metabolic, structural and histological functions of the gut microbiome [39], which has driven scientists to develop unique microbial signatures that describe IBD [4]. Further analysis of the interaction between the microbiome and other MMPs upregulated in IBD [1-3,8,12] are required to yield further insight into microbe-microbe and host-microbe interactions.

## Methods

### Bacterial strains and growth conditions

*C. rodentium,* strain DBS 100 (generously provided by the late Dr. David Schauer, Massachusetts Institute of Technology, Cambridge, MA) was grown on Luria-Bertani (LB) agar plates overnight at 37°C, followed by overnight culture in LB broth at 37°C without shaking, yielding a final bacterial concentration of approximately 10^9^ colony-forming units (CFU)/mL.

### Mouse strains and bacterial infection

Male and female wild-type (C57BL/6 J) and MMP-9^−/−^ (B6.FVB(Cg)-*Mmp9*^*tm1Tvu*^/J) mice aged 5–6 weeks were purchased (Jackson Laboratory, Bar Harbour, ME) and housed in the containment unit of Laboratory Animal Services at the Hospital for Sick Children in cages containing a maximum of 5 mice per cage. All mice were allowed free access to food and water (supplied from a controlled source) for the duration of the study protocol. Animals were allowed to acclimatize for a period of one week prior to the start of the treatment protocols. *C. rodentium* (10^8^ CFU in 0.1 mL) was administered by orogastric gavage [40]. Sham animals were challenged with an equal volume of sterile LB broth. Mice were infected on day 0 (0d), weighed daily and sacrificed at either 10d or 30d post-infection. All experimental procedures were approved by the Hospital for Sick Children’s Animal Care Committee.

### Western blotting and gelatin zymography

Segments of distal colon were collected and homogenized in RIPA buffer (1% Nonidet P-40, 0.5% sodium deoxylate, 0.1% sodium dodecyl sulfate [SDS] in PBS) supplemented with 150 mM NaCl, 50 mM sodium fluoride, 1 mM sodium orthovanadate, 20 μg/mL phenylmethylsulfonyl fluoride, 15 μg/mL aprotinin, 2 μg/mL leupeptin, and 2 μg/mL pepstatin A (all from Sigma-Aldrich, Oakville, ON), and stored at −80°C. Protein was quantified in each sample by using the Bradford assay.

For immunoblotting, samples were loaded at a concentration of 25 μg of protein/well in 1x loading buffer and electrophoresed in 12% SDS polyacrylamide gels (Bio-Rad, Mississauga, ON) at a constant voltage of 120 V until resolution of the MMP-9 band was achieved. To verify equivalent samples, mouse monoclonal anti-β-actin (1:5,000; Sigma, St. Louis, MO) was used as a loading control. Gel proteins were transferred at 4°C onto nitrocellulose membranes at 250 mA for 150 min. Membranes were washed in Tris buffered saline (Sigma-Aldrich) and blocked in Odyssey blocking buffer (Leica, Toronto, ON) for 1 hr at room temperature. The membrane was incubated with primary antibody (anti-β-actin (1:5000) [Sigma-Aldrich]; anti-MMP-9 (1:1000) [Abcam, Cambridge, MA] diluted in Odyssey blocking buffer containing 0.1% Tween-20 (Od-T) overnight at 4°C. The membrane was then washed in TBS containing 0.1% Tween-20 (TBS-T), blocked for 1 hr in Od-T containing 1% donkey serum (Jackson Immunoresearch, West Grove, PA) and treated with relevant IR-dye-conjugated donkey secondary antibody (Rockland, Gilbertsville, PA) in Od-T for 1 hr at room temperature. After washing in TBS-T, immunoreactivity was visualized using an infrared imaging system (Odyssey) with 700 and 800 nm channels at a resolution of 169 μm (LI-COR Biosciences, Lincoln, NE).

Gelatin zymography was performed by diluting colonic homogenates in zymogram sample buffer (Bio-Rad) and electrophoresing the samples in precast 10% SDS-poly-acrylamide gels with gelatin (Bio-Rad) at 120 V until resolution was achieved. Gels were removed from their casings, gently rinsed in ddH2O, and placed onto a shaker in 1X renaturation buffer (Bio-Rad) for 1 hr, changing the buffer once at 30 mins. Gels were then placed in 1X development buffer (Bio-Rad), incubated at 37°C overnight and stained with Page Blue (Fermentas, Burlington, ON) for 1 hr before destaining in water for 1 hr and imaging on a Li-Cor Odyssey system.

### FITC-dextran permeability assay

Intestinal epithelial barrier function was measured *in vivo* using a 4 kDA fluorescein isothiocyanate-dextran probe (FD4, Sigma-Aldrich, Oakville, ON, Canada) measured in serum, as previously described [8]. FITC-dextran serum concentrations were determined by fluorometry (Perkin Elmer, Woodbridge, ON, Canada).

### Histology and immunocytochemistry

Distal segments of colon [9] were excised following sacrifice, gently scraped to remove fecal material, fixed in 10% neutral-buffered formalin and embedded in paraffin blocks. Tissue was sectioned at 4 μm thickness and stained with haematoxylin and eosin. Sections were visualized on a Leica DMI 6000B microscope using Leica Application Suite Advanced Fluorescence 2.2.1 software (Leica). Crypt depths were measured on coded sections by a blinded observer (DMR) using Leica Image Manager 500 software (Leica). Final crypt measurements per animal represent the average of 10 crypt lengths per section of tissue from two non-adjacent colonic sections.

Colonic sections from sham and *Citrobacter rodentium*-infected mice (day 10) were used for immunocytochemical examination of MMP-9 expression and localization. Briefly, 5μm-thick paraffin-embedded sections were deparaffinized in citroclear (National Diagnostics, Atlants, GA, USA), and rehydrated in graded concentrations of ethanol. The antigen was exposed by steaming sections for 30 min in 10 mM citrate buffer (pH 6.0)/0.05% Triton X-100 (VWR, Mississauga, ON). Sections were then blocked in 3% bovine serum albumin (Sigma-Aldich), and incubated with either a polyclonal anti-MMP-9 antibody (1:200) or a rabbit primary antibody (Rb) isotype control (Invitrogen, Burlington, ON) overnight at 4°C. Sections were then washed in PBS and incubated with AlexaFluor®488 goat anti-rabbit IgG (1:400; Invitrogen), stained with DAPI (1:36,000, Invitrogen) and mounted onto slides with fluorescence mounting medium (Dako, Burlington, ON). Fluorescence was visualized on a Leica DM16000B (Leica, Concord, ON) equipped with a DFC360FX monochromatic camera (Leica). Leica Application Suite imaging software was used for all analyses and images recorded at identical gain settings.

Periodic acid Schiff staining was used to demonstrate the presence of mucin-containing vacuoles indicative of goblet cells [41]. Following the Aldrich Periodic Acid-Schiff (PAS) Staining System (Procedure No. 395, Sigma), colonic samples were de-paraffinized and oxidized in 0.5% periodic acid for 5 min. Slides were then rinsed in distilled water, placed in Schiff reagent, washed, counterstained in Mayer’s hematoxylin, mounted onto slides and then visualized microscopically. Ten well oriented crypts per section of distal colon from each animal were assessed, using coded slides, for numbers of PAS-positive stained cells per crypt.

### qPCR analysis of pro- and anti-inflammatory markers

Full-thickness distal colons were homogenized in Trizol (Invitrogen, Burlington, ON, Canada) and RNA extracted using a phenol-chloroform extraction protocol (Invitrogen). To eliminate DNA contamination, DNAse A (Invitrogen) was used, according to the manufacturer’s instructions. cDNA libraries then were generated using an iSCRIPT cDNA synthesis kit (Bio-Rad), and subsequently amplified by quantitative PCR using SSO Fast EvaGreen Supermix and a CFX96 C1000 Thermal Cycler (BioRad). Primers against mouse β-actin (housekeeping gene), IL-4, IL-10, IL-17α, TNFα, IFNγ and Foxp3 (Table 3) were utilized, as described previously [42].

**Table 3:** Mouse primers employed in this study

**Gene**	**Forward primer (5’ to 3’)**	**Reverse primer (5’ to 3’)**
β-actin	CCAGTTGGTAACAATGCCATGT	GGCTGTATTCCCCTCCATCG
IL-4	GCCGATGATCTCTCTCAAGTGA	GGTCTCAACCCCCAGCTAGT
IL-10	CGCAGCTCTAGGAGCATGTG	GCTCTTACTGACTGGCATGAG
IL-17α	CTTTCCCTCCGCATTGACAC	TTTAACTCCCTTGGCGCAAAA
TNFα	GCTACGACGTGGGCTACAG	CCCTCACACACTCAGATCATCTTCT
IFNγ	CCATCCTTTTGCCAGTTCCTC	ATGAACGCTACACACTGCATC
Foxp3	ACCACACTTCATGCATCAGC	ACTTGGAGCACAGGGGTCT

### Gut microbiome analysis

Fecal pellets were collected from mouse colons after animal sacrifice and stored at −80°C. DNA was extracted using the QIAamp DNA stool kit (QIAGEN, Toronto, ON), according to the manufacturer’s instructions. The fecal microbiome was studied in wild-type (WT) and MMP-9^−/−^ infected and non-infected mice using two complementary techniques.

For a holistic view of the microbiome structure, terminal restriction fragment length polymorphism (T-RFLP) was used to assess evenness and the Shannon-Weiner diversity index. Briefly, as previously described [21], DNA was extracted from each individual mouse and quantified using a NanoDrop 2000c spectrophotometer (Thermo Scientific, New York, NY). PCR amplification was run in duplicate for each sample with 8 F and 1492R primers. Agarose gel electrophoresis was used to purify the sample and a band at approximately 1.6 kb was excised and purified using a gel extraction kit (Qiagen, Mississauga, ON). DNA was digested with MspI (New England Biolabs Inc., Pickering, ON) for 30 mins at 37°C and subject to capillary electrophoresis using an ABI 3130 Genetic Analyzer. Electropherograms were generated from individual mice and *C. rodentium* colonization monitored by identifying and quantifying a 118 bp digested fragment length unique to *C. rodentium*. NMS was carried out on terminal restriction fragments using PC-ORD Version 6.0 (MjM Software Design, Oregon, USA Sørensen (Bray-Curtis) was used as the distance measure and random starting configurations were used with 250 runs of real data. The final stress of the best solution was 10.6, with three dimensions in the final solution. The Monte Carlo test used 249 randomized runs and produced a *p*-value of 0.0040. Multi-response permutation procedure (MRPP) was used to compare differences between experimental groups by analysis of the chance-corrected within group agreement (A) and *p*-value [43].

qPCR was used for a reductionist view of specific bacterial communities (Bacilli, Bacteroides, Enterobacteriaceae, Firmicutes, Lactobacillus, and segmented filamentous bacteria) utilizing previously published primers and protocols [42].

### Statistical analyses

Results are expressed as means, +/− standard error of the mean (SEM). Comparisons were performed between multiple experimental groups by using either 2-way analysis of variance (ANOVA) or Student’s t-test, where indicated. P values of < 0.05 were considered significant.

## Authors’ contributions

DMR carried out *in vivo* work, western blotting and gelatin zymography. AJS carried out the microbiome analysis. LV and SAK conducted the immunocytochemistry. DMR, AJS, SPH, LV, MGG, SAK, KCJH, and PMS conceived of the study, participated in its design and coordination and writing of the manuscript. All authors read and approved the final manuscript.

## Authors’ information

PMS is a Senior Scientist in the Cell Biology Program at the Hospital for Sick Children, and Professor of Paediatrics, Laboratory Medicine and Pathobiology and Dentistry at the University of Toronto. PMS holds a Canada Research Chair (tier 1) in Gastrointestinal Disease.
